# A case of reversible drug‐induced liver failure

**DOI:** 10.1002/ccr3.1030

**Published:** 2017-06-02

**Authors:** Mohammad Ansari, Sabrina Arshed, Mohammed Islam, Shuvendu Sen, Abdalla Yousif

**Affiliations:** ^1^ Department of Internal MedicineRaritan Bay Medical CenterPerth AmboyNew Jersey

**Keywords:** Cocaine, dialysis, kidney failure, liver failure, N‐acetylcysteine

## Abstract

Acute fulminant liver failure and acute renal failure are devastating complications caused by many drugs. The use of N‐acetylcysteine has been well established in acetaminophen toxicity, but it remains controversial in other cases. Dialysis is a very effective method of removing certain drugs from the system. With the invention of new street drugs such as “synthetic marijuana,” it may be beneficial in patients whom the substances ingested are unknown. We report a case of a 42‐year‐old male who developed acute fulminant hepatic failure and acute renal failure, who was cured with dialysis, N‐acetylcysteine, and other supportive measures.

## Introduction

About 6.4 million people in North America use cocaine, accounting for approximately 45% of global use 1. In literature, its effects on the cardiac, cerebrovascular, pulmonary, and gastrointestinal systems are well known and reported by the medical community 1. The effects and management of cocaine on the liver and kidney are not as well reported 1. Current guidelines recommended discontinuation of the offending agent, supportive measures, and if necessary liver transplant 2, 3. The role N‐acetylcysteine (NAC) is well established in acetaminophen toxicity, but not so much in regard to other substances 2, 3. Dialysis is a very effective method in removing certain substances from the body. However, it is often unknown what a patient has ingested. We report a case of a 42‐year‐old male with a history of polysubstance use brought by EMS to the hospital because of unusual behavior. On our initial evaluation, he was found to have cocaine and THC in his system, along with liver and renal failure.

## Case Presentation

A 42‐year‐old male, with a past medial history of polysubstance abuse and major depressive disorder, was brought by EMS after a call was placed by the patient's tenant that the patient was destroying his own apartment. When the EMS arrived, the patient was very lethargic and unable to communicate. Marijuana was found in the apartment. He was given two doses of Narcan which did not affect him. According to the patient's brother, he was last seen in his usual state of health 2 days prior. His vitals were blood pressure 106/70 mmHg, pulse 135/min, respiratory rate 20/min, temperature 100.7 Fahrenheit, and pulse oximetry on room air 93%. On examination, he was very lethargic, confused, and unable to form any comprehensible sentences. He tested positive for cocaine and THC. Serum acetaminophen and alcohol levels were negative. His initial laboratory tests showed a bicarbonate level of 18, BUN of 27, creatinine of 4, GFR of 17, anion gap of 32, INR of 3.1, lactic acid of 13.3, creatine kinase of 11731, AST of 1111, ALT of 869, and total bilirubin of 1.1. An abdominal ultrasound showed no urinary tract obstruction or liver abnormalities. He was initially given intravenous fluids for the acute renal failure, but his creatinine progressively got worse and peaked at 8 mg/dL. Dialysis was started on day 3, after which the patient's mental status improved significantly. The patient's AST/ALT reached above 2000, total bilirubin was 3.6, and INR was 5.1. On day 3, NAC was started and the patient was transferred to another facility to be managed by a hepatologist. Testing for other causes of acute liver failure, including hepatitis a/b/c, atypical alcohols, and autoimmune panels came back negative. A week later, NAC and dialysis were discontinued as his liver and kidney functions had significantly improved. Liver biopsy was never performed as the patient's liver function responded to medical treatment. The patient followed up in our outpatient clinic a month after his initial admission. The patient felt great and was unable to remember anything about his illness until after his first dialysis. Laboratory tests were performed which showed complete resolution of his kidney and liver injury (Table 1 shows all the laboratory tests imaging performed during the patient's hospital stay and as an outpatient).

**Table 1:** Laboratory tests and imaging

Diagnostic	Day 1	Day 2	Day 3	Day 32
BUN (mg/dL)	27	33	39	15
Creatinine (mg/dL)	4	5.2	8.0	0.9
Bicarbonate (mmol/L)	18	13	16	22
GFR (mL/min/1.73 m^2^)	17	13	8	>60
AST (U/L)	1111	1516	2137	37
ALT (U/L)	869	1005	2798	42
INR	3.1	4.9	5.1	0.9
Total bilirubin (mg/dL)	1.1	1.4	3.6	0.8
CK (IU/L)	11,731	10,738	5289	156
Urine drug screen	Cocaine, THC			Negative
Alcohol level (mg/dL)	<10			
Abdominal ultrasound	Normal			
Atypical alcohols	Negative			
Hepatitis A IGM	Negative			
Hepatitis B surface antibody	Negative			
Hepatitis B core antibody	Negative			
Hepatitis C antibody	Negative			
ANA	Negative			
Antismooth muscle antibody	Negative			
Antimitochondrial antibody	Negative			
Liver biopsy	Not performed			

## Discussion

It is well established that NAC has been proven to decrease the burden of acetaminophen toxicity on the liver by replenishing glutathione which converts toxic metabolites (NAPQI) into benign products 2, 3, 4. It also has the ability to scavenge oxygen‐derived free radicals and improve endothelium‐dependant perfusion to the liver 2, 3, 4. Like acetaminophen, cocaine also has toxic metabolites (norcocaine and N‐hydroxynorcocaine) that cause liver damage 1, 5, 6. Cocaine and its metabolites cause increased lipid peroxidation, free radical activity, and impaired calcium sequestration 5. NAC's role has not been well established as an antidote for nonacetaminophen drug‐induced (NADI) liver failure. A prospective trial showed significantly improved transplant‐free survival at 3 weeks and at 1 year (52% vs. 30%) with the use of NAC for the treatment of NADI liver failure 2, 7, 8. Another trial showed that NAC was associated with a statistically significant survival benefit (47% vs. 27%) 2, 7, 8. In a case series of patients with drug‐induced liver injury secondary to Amanita phalloides mushroom poisoning, 10 of 11 patients recovered fully after receiving NAC with supportive measures 3. Current guidelines do not include NAC for NADI liver failure. Many physicians will start NAC in acute liver failure because it is relatively benign and has been shown to improve patients’ outcome in NADI liver failure in several studies 9. We strongly believe NAC should be a part of the treatment for any acute liver failure, at least until, drugs have been ruled out as the possible cause of the original insult.

Refractory to initial forced diuresis with intravenous fluids, our patient's acute renal failure was eventually managed with dialysis. Immediately after the patient's first dialysis session, the patient's mental status improved. The mechanism if acute renal failure by cocaine is thought to be secondary to intense renal vasoconstriction and rhabdomyolysis 1. Although it is established that cocaine and its metabolites are not dialyzable, it addresses another issue 1, 7. There is a pandemic of new street drugs such as “synthetic marijuana” that have emerged into the market. Many of these drugs cannot be tested, and if so take a great deal of time to be analyzed 4. We are unaware of all their complications, and the clinical outcome is very unpredictable 4. This patient was known to be a polysubstance abuser from previous medical records. Perhaps he took some other drugs which we never found out about. His mental status and liver function improved immediately after dialysis was started. We believe that dialysis should have been initiated earlier in our patient. Dialysis can serve to detoxify the body of drugs when we are unsure about what the patient took in the first place. Perhaps dialysis should be a routine part of management in such situations, as the benefits outweigh the risks.

## Conclusion

In conclusion, our patient with acute fulminant liver failure and acute renal failure made a full recovery. Further research needs to be carried out on NACs role to neutralize nonacetaminophen drug‐induced toxicity. NAC is a relatively benign drug which can be given empirically, as it is seen to improve outcome in such cases. With the invention of new street drugs, at times we are unable to determine what has been ingested. Dialysis should be promptly initiated in such cases of uncertainty as the benefits outweigh the risks.

## Authorship

MA, first author: did all research, wrote the manuscript, and took care of patient. SA: gave ideas on how to approach paper and edited grammar and content on manuscript. MI: took care of patient with first author and was the attending physician. SS: gave ideas on how to approach paper and edited the manuscript for content and grammar. AY: helped take care of patient and reviewed final manuscript for content and grammar.

## Conflict of Interest

None declared.
